# Clinical Notes on Characteristics

**Published:** 1888-01

**Authors:** 


### CLINICAL NOTES ON CHARACTERISTICS.

Our readers will miss this month's installment of these notes. The reason is that our space is so crowded that we were obliged to defer them until the February number.

Several other articles similarly have been laid aside for want of room.

We invite the freest criticism of these notes. Any corrections or additions will be gladly accepted and incorporated, our intention being to republish them in book form when finished.

It is desired that all communications on the subject shall be in form suitable for publication in our pages.

